# Supplementary material for: Analyses of cancer incidence and other morbidities in neutron irradiated B6CF1 mice
Source: PLoS One. 2021 Mar 3;16(3):e0231511. doi: 10.1371/journal.pone.0231511 (PMC7928494; doi:10.1371/journal.pone.0231511)
Supplement: S1 File — (PDF) [file pone.0231511.s001.pdf]

Analyses of cancer incidence and other morbidities in neutron irradiated B6CF1 mice.

Alia Zander[1], Tatjana Paunesku[1], Gayle Woloschak[1]

[1]Northwestern University, Feinberg School of Medicine, Radiation Oncology, Chicago, IL

S1 Fig: Controls analysis with grouped fractions

A

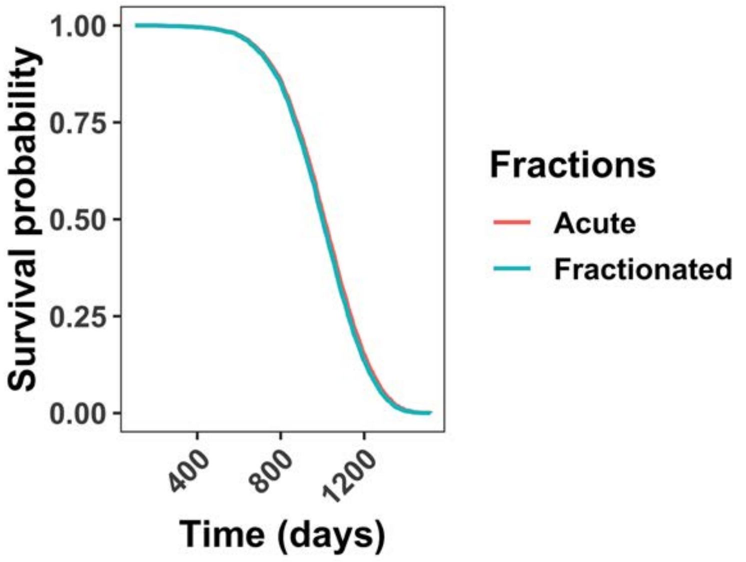

C

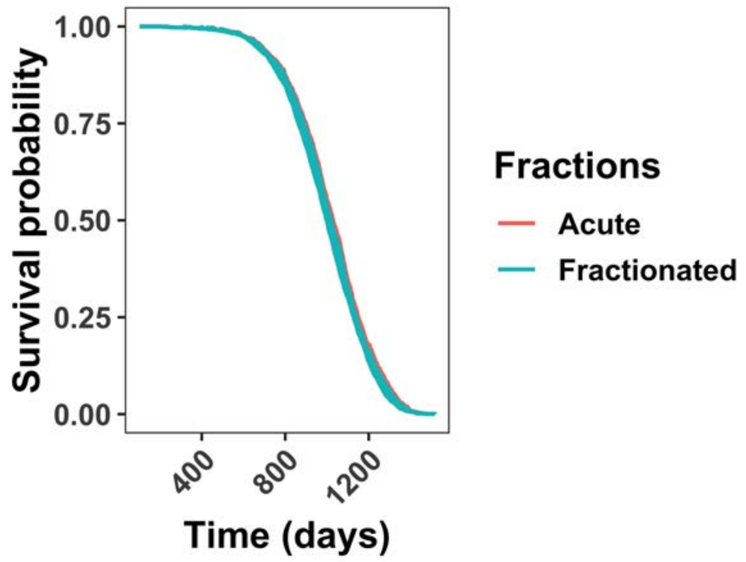

E

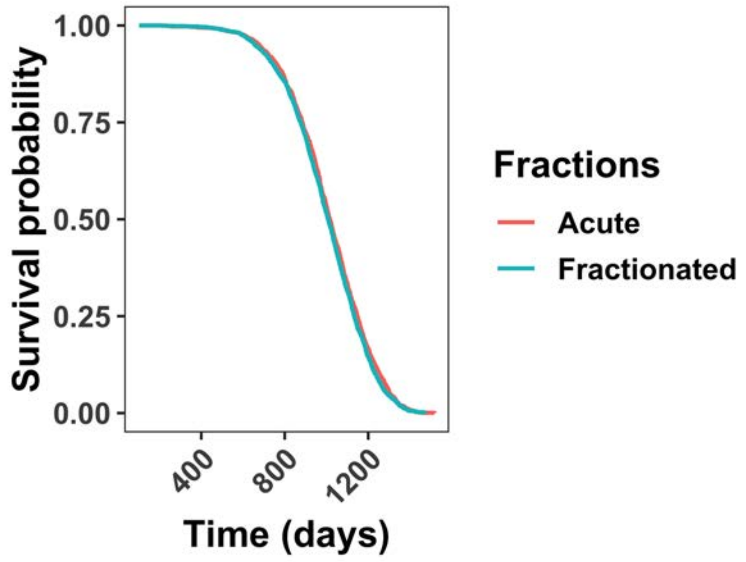

B

Term	Estimate	Hazard Ratio (95% CI)	P-value
Sex(M)	-0.103	0.902 (0.84, 0.96)	**0.002**
Fractionated	0.054	1.055 (0.99, 1.13)	0.099

D

Term	Estimate	Hazard Ratio (95% CI)	P-value
Fractionated	0.054	1.055 (0.990, 1.125)	0.098

S1 Table: Data filtering for neutron analysis

Data removed	Reasoning	# of mice
-	-	50,110
JM11	Not a true data set	49,225
JM10	Different species – Peromyscus leucopus	46,835
JM14 mice treated with radioprotectors	Beyond the scope of our project	46,635
Breeder mice	Held under different conditions	43,428
JM2 mice	Held under different conditions	31,843
COD – removal to another experiment	Mice listed under different experiment, do not want to double count	28,153
JM12 mice	Controls analysis showed significant difference	27,553
JM3 mice	Controls analysis showed significant difference	24,478
Mice irradiated with 300 fractions	Controls analysis showed significant difference	23,953
JM8 mice	Experimental design – separate analysis required	22,626
Gamma irradiated mice	Different quality of radiation - separate analysis	14,778
Mice irradiated with 120 fractions	Neutron irradiated mice never received 120 fractions	14,578

S2 Table: Mixed effects model applied to control animals

Term	Estimate	Hazard Ratio	P-value
Sex(M)	-0.210	0.810 (0.77,0.85)	<0.001
Fractionated	0.023	1.023 (0.88, 1.18)	0.76
Total Dose	0.019	1.019 (1.01, 1.02)	<0.001
Age First Irradiated	-0.002	0.998 (0.99, 1.00)	0.23
Fractionated:Total Dose	-0.010	0.990 (0.99, 0.99)	<0.001

S3 Table: COD toxicities

Cause of Death	Total	Control			Gamma			Neutron		
		Total	Female	Male	Total	Female	Male	Total	Female	Male
Abscess	4	1	0	1	2	0	2	1	1	0
Acute infection	65	6	1	5	25	9	16	34	15	19
Adhesion	3	0	0	0	3	2	1	0	0	0
Anemia	780	48	32	16	402	204	198	330	168	162
Aneurysm	1	0	0	0	0	0	0	1	1	0
Ascites	4	1	1	0	1	1	0	2	0	2
Bacteremia	10	0	0	0	8	3	5	2	2	0
Bloody hydrothorax or ascites	7	1	1	0	3	1	2	3	3	0
Urinary bladder	7	0	0	0	3	1	2	4	4	0
Bone	1	0	0	0	1	0	1	0	0	0
Brain	27	3	2	1	11	9	2	13	12	1
Caecum	3	0	0	0	2	2	0	1	1	0
Cause of death unknown	1398	253	150	103	488	246	242	657	393	264
Cowper’s gland	2	0	0	0	1	0	1	1	0	1
Cholecystitis	2	0	0	0	0	0	0	2	1	1
Cirrhosis	14	4	2	2	3	2	1	7	4	3
Calculi	1	0	0	0	1	1	0	0	0	0
Clear hydrothorax or ascites	1	0	0	0	0	0	0	1	1	0
Central nervous system	12	2	2	0	3	0	3	7	5	2
Colon	2	2	2	0	0	0	0	0	0	0
Chronic renal disease	55	13	9	4	17	12	5	25	20	5
Cyst	103	19	18	1	43	40	3	41	39	2
Dermatitis	10	3	1	2	3	1	2	4	4	0
Diverticulum	304	56	41	15	128	63	65	120	75	45
Duodenum	1	0	0	0	1	0	1	0	0	0
Edema	2	0	0	0	1	0	1	1	0	1
Enteritis	206	46	21	25	84	30	54	76	49	27
Esophagus	2	0	0	0	1	0	1	1	1	0
Fighting	6	0	0	0	0	0	0	6	0	6
Gallbladder	2	2	2	0	0	0	0	0	0	0
Hematoma	1	0	0	0	1	1	0	0	0	0
Hepatitis	89	11	6	5	30	18	12	48	25	23
Hydronephrosis	140	35	33	2	43	39	4	62	56	6
Hemorrhage	202	40	28	12	63	33	30	99	65	34
Heart	15	4	2	2	7	2	5	4	2	2
Hydrothorax	15	0	0	0	6	3	3	9	3	6
Ileum	1	1	0	1	0	0	0	0	0	0
Inflammation	14	3	2	1	4	3	1	7	4	3
Intussusception	1	0	0	0	0	0	0	1	1	0
Jejunum	2	1	1	0	1	0	1	0	0	0
Kidney	2	0	0	0	1	0	1	1	0	1
Liver	50	6	4	2	17	7	10	27	20	7
Malocclusion	7	0	0	0	3	2	1	4	4	0
Metritis	24	4	4	0	0	0	0	20	20	0
Megacolon	2	0	0	0	0	0	0	2	0	2
Miscellaneous circulatory	4	1	0	1	1	0	1	2	1	1
Miscellaneous digestive	7	3	3	0	1	0	1	3	1	2
Miscellaneous urogenital	37	9	0	9	13	0	13	15	1	14
Miscellaneous lung disease	3	0	0	0	0	0	0	3	1	2
Miscellaneous renal (urinary tract)	20	3	1	2	9	2	7	8	2	6
Others, general	9	2	1	1	6	2	4	1	0	1
Milky	5	0	0	0	3	3	0	2	1	1
Necrosis	3	0	0	0	1	1	0	2	1	1
Non-thymic lymphoma, generalized	7937	1729	1119	610	2747	1403	1344	3461	2229	1232
Non-thymic lymphoma, localized	298	61	40	21	105	57	48	132	93	39
Obstruction	8	0	0	0	4	1	3	4	4	0
Ovary	5	0	0	0	2	2	0	3	3	0
Paralysis	3	0	0	0	0	0	0	3	2	1
Pericardium	14	1	1	0	3	1	2	10	6	4
Polycystic kidney disease	3	1	1	0	2	0	2	0	0	0
Peritonitis	19	1	1	0	6	4	2	12	10	2
Pneumonitis	325	56	46	10	89	65	24	180	143	37
Pneumonia	603	93	69	24	183	122	61	327	254	73
Perforation	3	0	0	0	0	0	0	3	3	0
Prolapse	7	2	2	0	3	2	1	2	2	0
Seminal vesicle	1	0	0	0	1	0	1	0	0	0
Salivary gland	1	0	0	0	1	1	0	0	0	0
Spleen	10	2	0	2	2	1	1	6	4	2
Stomach	1	0	0	0	0	0	0	1	1	0
Lung tumor	4392	859	296	563	1471	366	1105	2062	672	1390
Adipose tumor	1	0	0	0	0	0	0	1	1	0
Adrenal tumor	85	8	3	5	27	20	7	50	27	23
Urinary bladder tumor	9	4	2	2	0	0	0	5	3	2
Bone tumor	153	14	12	2	31	19	12	108	86	22
Brain tumor	31	2	1	1	12	8	4	17	12	5
Caecum tumor	9	2	2	0	4	2	2	3	3	0
Cowper’s gland tumor	1	0	0	0	1	0	1	0	0	0
Central nervous system tumor	3	0	0	0	2	1	1	1	1	0
Colon tumor	15	3	2	1	3	2	1	9	7	2

S3 Table, continued: COD toxicities

Connective tissue tumor (fibrosarcoma)	462	105	90	15	116	89	27	241	200	41
Duodenum tumor	4	1	0	1	0	0	0	3	2	1
Testis and epididymis	1	0	0	0	0	0	0	1	0	1
Epididymis tumor	6	0	0	0	1	0	1	5	0	5
Esophagus tumor	1	1	1	0	0	0	0	0	0	0
Harderian glad tumor	190	11	5	6	30	12	18	149	74	75
Hibernating gland tumor	3	1	1	0	0	0	0	2	2	0
Thrombos	43	10	3	7	19	5	14	14	9	5
Heart tumor	15	3	0	3	7	2	5	5	4	1
Ileum tumor	9	2	1	1	3	1	2	4	1	3
Jejunum tumor	17	2	1	1	9	3	6	6	1	5
Kidney tumor	170	9	6	3	57	25	32	104	42	62
Liver tumor	394	72	23	49	115	36	79	207	105	102
Mammary gland tumor	54	17	17	0	12	12	0	25	25	0
Miscellaneous connective tissue tumor	35	6	4	2	16	9	7	13	9	4
Miscellaneous digestive system tumor	3	1	1	0	0	0	0	2	2	0
Miscellaneous glandular tumor	21	6	6	0	5	1	4	10	7	3
Miscellaneous lung tumor	46	10	8	2	19	12	7	17	12	5
Miscellaneous nervous system tumor	6	1	1	0	1	1	0	4	3	1
Miscellaneous reticular system tumor	4	1	1	0	3	1	2	0	0	0
Miscellaneous urologenital tumor	16	2	1	1	3	2	1	11	2	9
Muscle tumor	106	6	5	1	44	30	14	56	32	24
Ovary tumor	454	75	75	0	180	180	0	199	199	0
Pancreas tumor	14	0	0	0	4	4	0	10	8	2
Pituitary tumor	77	14	14	0	20	14	6	43	33	10
Preputial gland	1	0	0	0	0	0	0	1	0	1
Prostate tumor	9	3	0	3	3	0	3	3	0	3
Pylorus tumor	39	5	3	2	5	2	3	29	16	13
Secondary tumor	10	2	2	0	3	1	2	5	4	1

Salivary gland tumor	29	3	2	1	11	4	7	15	10	5
Skin tumor	6	1	1	0	2	0	2	3	3	0
Seminal vesicle tumor	10	0	0	0	2	0	2	8	1	7
Spleen tumor	49	7	4	3	15	8	7	27	19	8
Stomach tumor	25	3	1	2	6	2	4	16	11	5
Thyroid tumor	20	3	2	1	5	3	2	12	8	4
Testis tumor	34	3	0	3	12	0	12	19	0	19
Thymic lymphoma, generalized	363	37	32	5	220	135	85	106	89	17
Thymic lymphoma, localized	176	9	7	2	134	74	60	33	25	8
Uterus tumor	112	23	23	0	21	21	0	68	68	0
Vaginal tumor	7	2	2	0	3	3	0	2	2	0
Vascular tumor	1149	184	97	87	466	212	254	499	264	235
Twister	2	1	1	0	0	0	0	1	0	1
Typhlitis	19	3	2	1	6	3	3	10	4	6
Ulcer	5	0	0	0	2	0	2	3	0	3
Uterus	6	1	1	0	0	0	0	5	5	0
Volvulus	1	0	0	0	0	0	0	1	1	0

S2 Fig: Robustness tests for main model

A

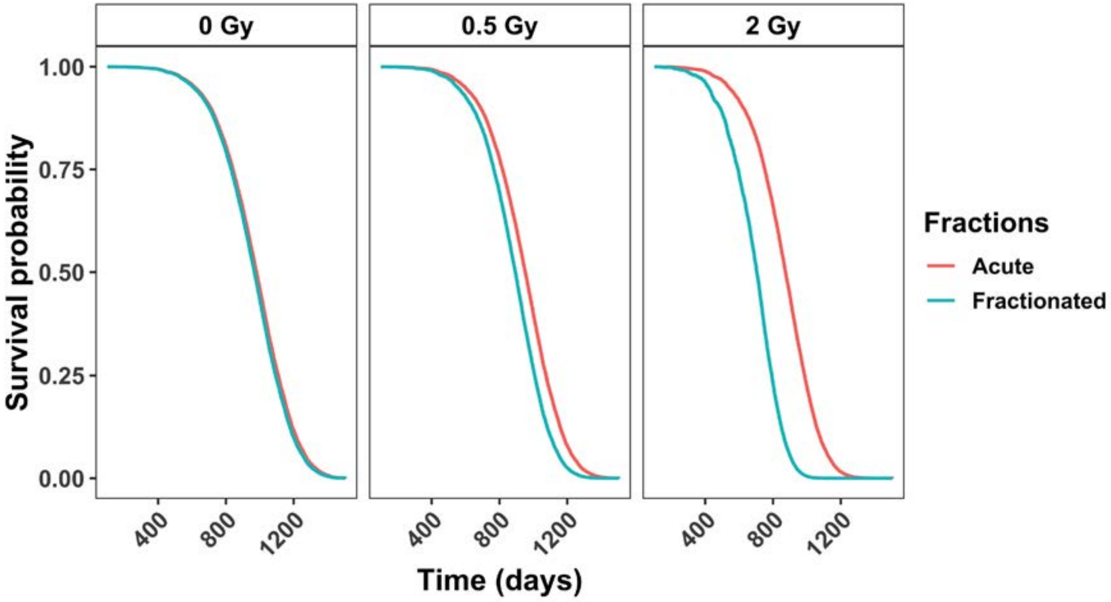

B

Term	Estimate	Hazard ratio (95% CI)	P-value
Sex(M)	-0.16	0.851 (0.814, 0.891)	**2.68E-12**
Fractionated	0.07	1.074 (1.073, 1.1336)	**0.010**
Total Dose	0.329	1.39 (1.19, 1.62)	**2.44E-05**
Age First Irradiated	0.0001	1.0001 (0.9996, 1.0006)	0.627
Fractionated:Total Dose	0.597	1.82 (1.55, 2.13)	**1.10E-13**

S2 Fig, continued: Robustness tests for main model

C

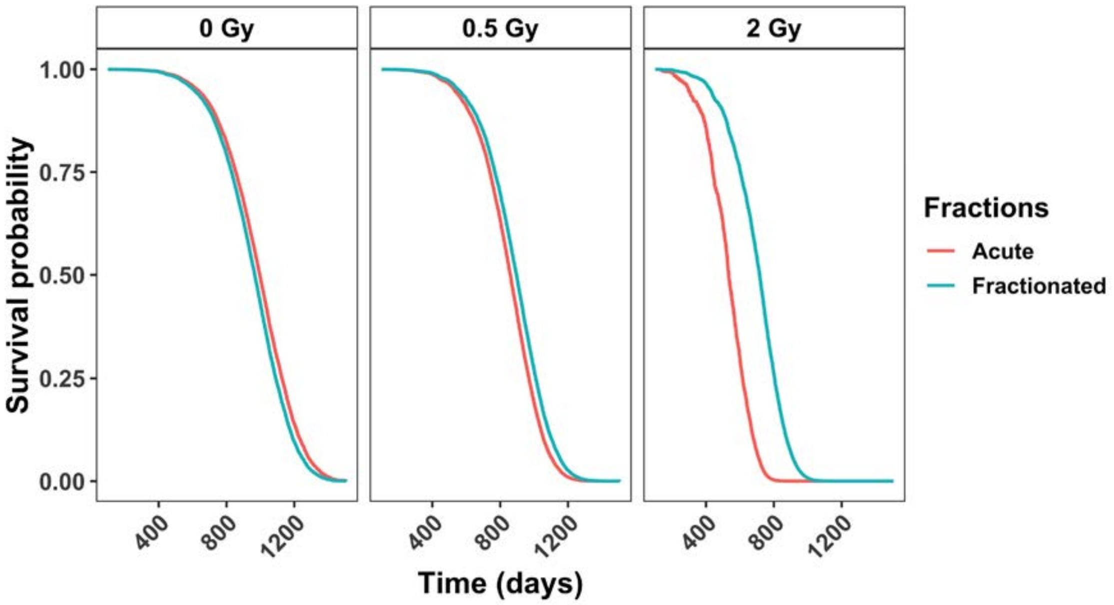

D

Term	Estimate	Hazard Ratio (95% CI)	P-value
Sex(M)	-0.17	0.843 (0.806, 0.882)	**1.18E-13**
Fractionated	0.175	1.191 (1.12, 1.27)	**4.00E-08**
Total Dose	2.167	8.73 (5.10, 14.94)	**2.56E-15**
Age First Irradiated	0.001	1.0009 (1.0004, 1.0014)	**0.0006**
Fractionated:Total Dose	-0.821	0.44 (0.29, 0.67)	**0.0002**
Total Dose:Age First Irradiated	-0.004	0.996 (0.995, 0.997)	**4.46E-12**

S2 Fig, continued: Robustness tests for main model

E

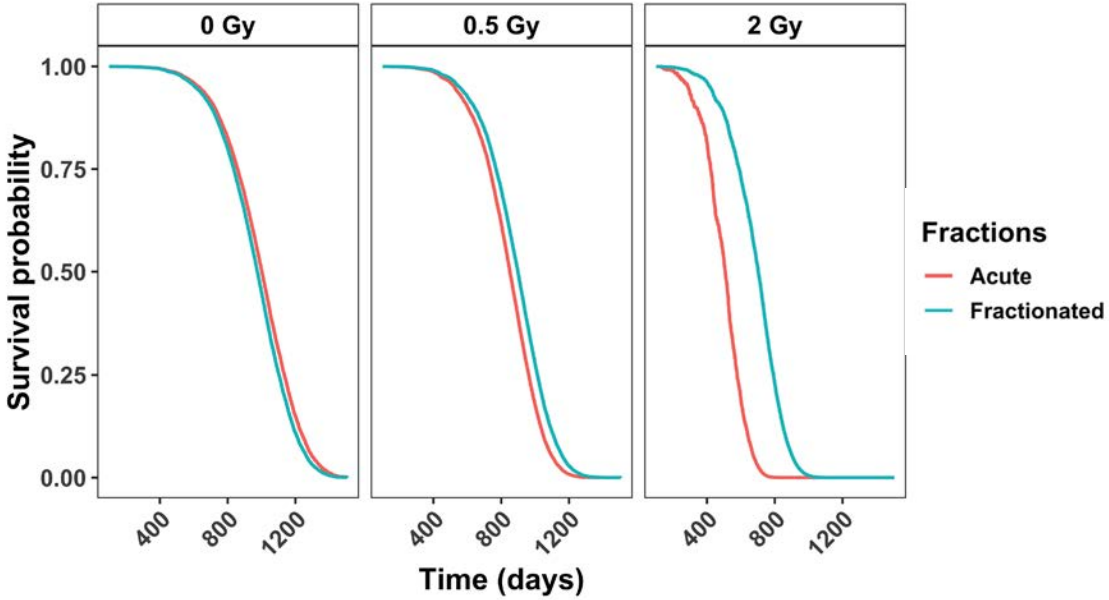

F

Term	Estimate	Hazard Ratio (95% CI)	P-value
Sex(M)	-0.19	0.83 (0.79, 0.87)	**3.19E-15**
Fractionated	0.154	1.17 (1.09, 1.25)	**7.83E-06**
Total Dose	1.581	4.86 (2.59, 9.126)	**8.58E-07**
Age First Irradiated	-0.0049	0.995 (0.991, 0.999)	**0.020**
Fractionated:Total Dose	-0.907	0.403 (0.26, 0.62)	**4.24E-05**
Total Dose:Age First Irradiated	2.25E-05	1.0000 (1.0000, 1.0001)	0.25

S2 Fig, continued: Robustness tests for main model

G

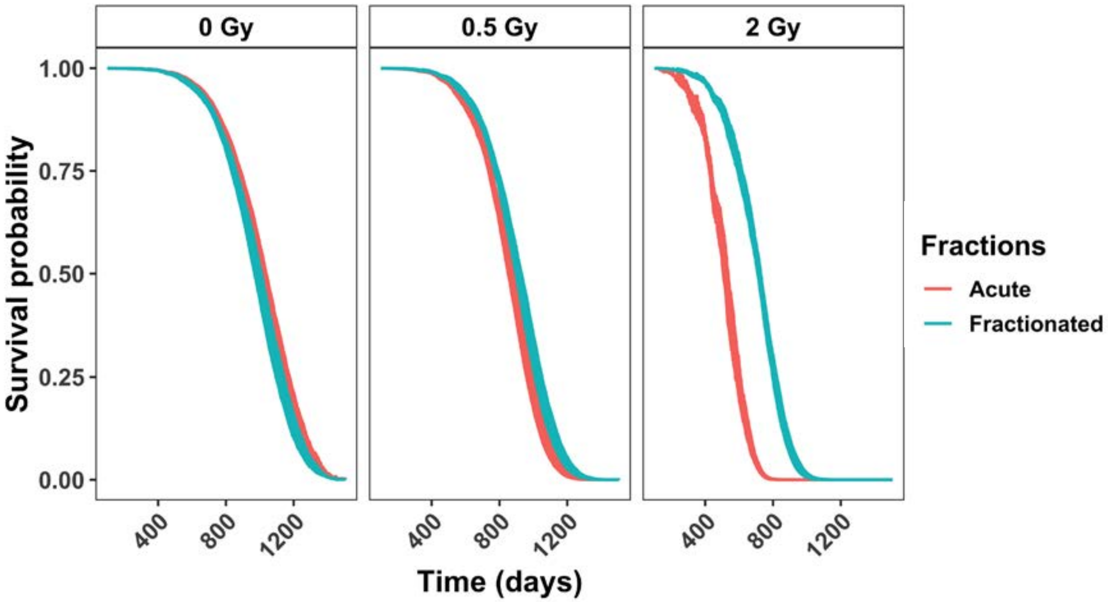

H

Term	Estimate	Hazard Ratio (95% CI)	P-value
Fractionated	0.16	1.177 (1.103, 1.257)	**1.12E-06**
Total Dose	1.826	6.21 (4.03, 9.57)	**1.18E-16**
Age First Irradiated	-0.003	0.997 (0.993, 0.9998)	**0.038**
Fractionated:Total Dose	-0.909	0.40 (0.26, 0.62)	**3.85E-05**

S2 Fig, continued: Robustness tests for main model

I

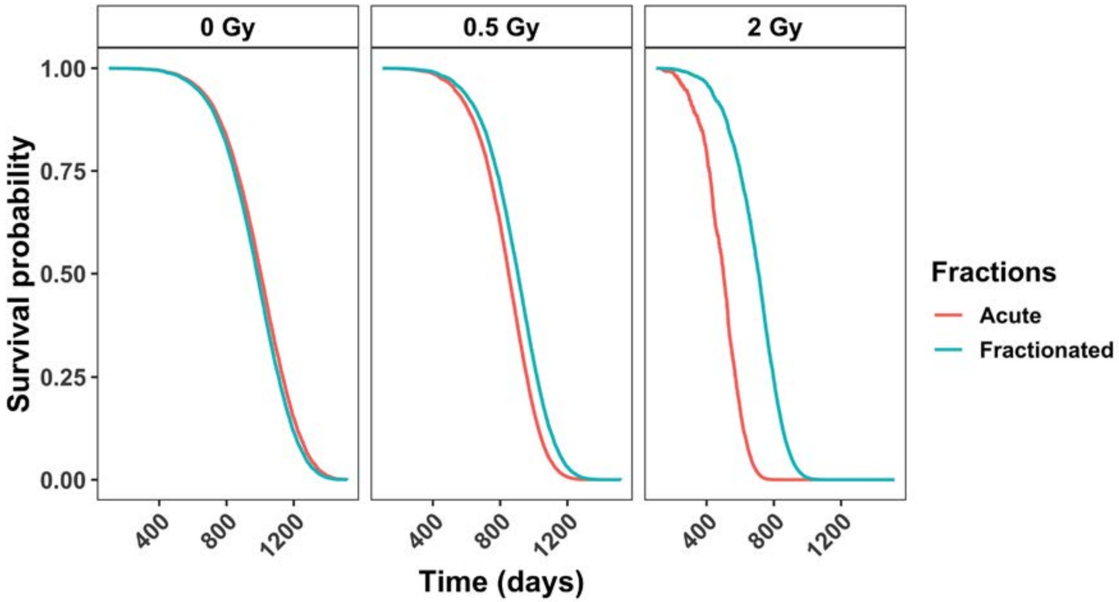

J

Term	Estimate	Hazard Ratio (95% CI)	P-value
Sex(M)	-0.153	0.86 (0.83, 0.89)	0
Fractionated	0.125	1.13 (1.08, 1.18)	0
Total Dose	1.93	6.89 (4.71, 10.08)	0
Age First Irradiated	-0.003	0.9975 (0.99, 1.00004)	0.053
Fractionated:Total Dose	-0.967	0.38 (0.26, 0.56)	0

S3 Fig: KM curves for control and neutron irradiated mice to validate Cox PH model

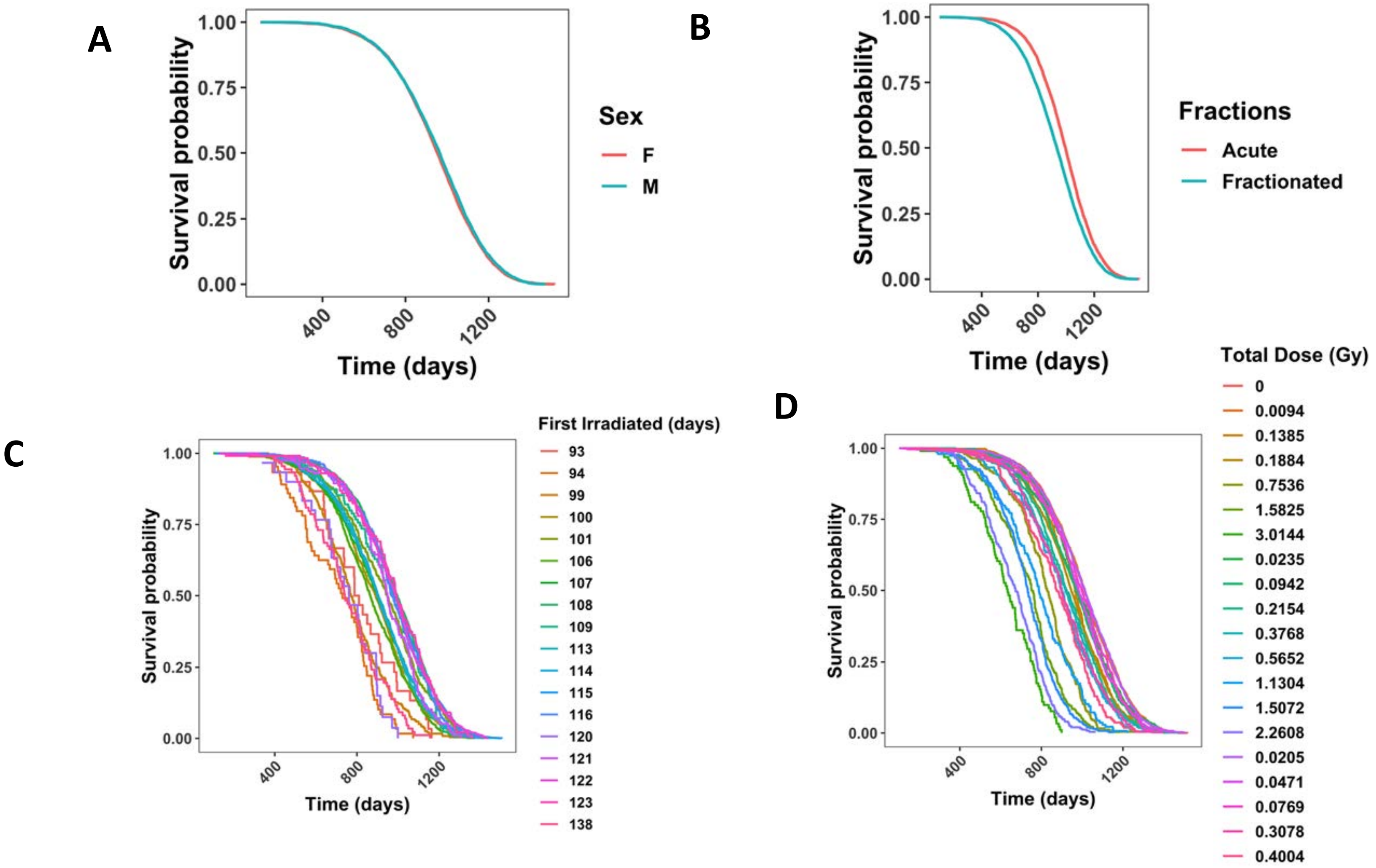

S4 Fig: Cause specific hazards for lung tumors, non-thymic lymphomas, and tumors (excluding lung tumors)

A

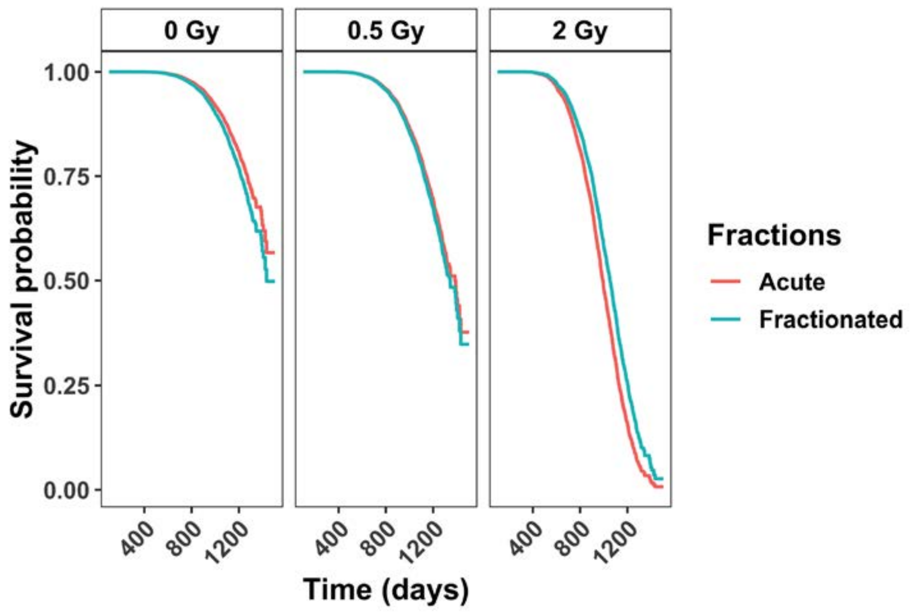

B

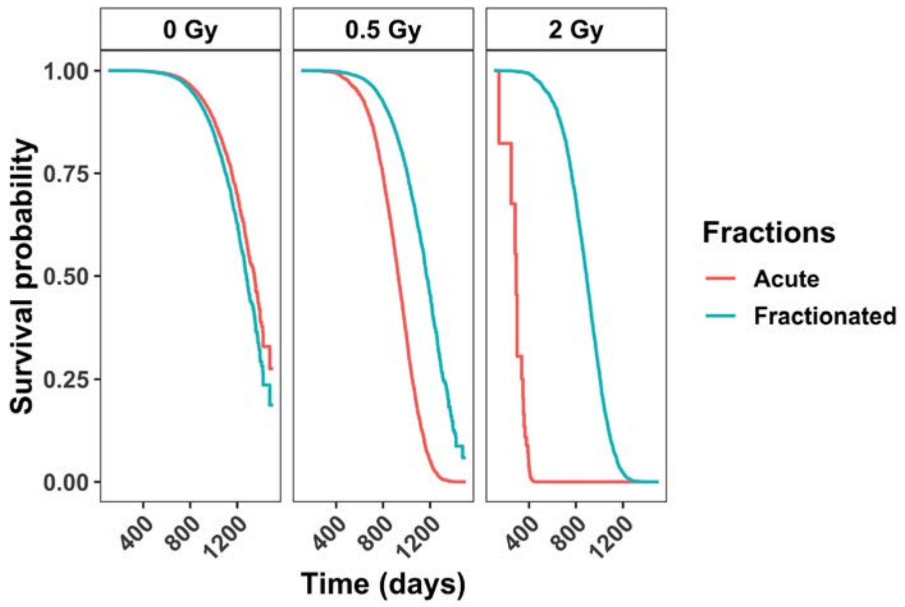

S4 Fig, continued: Cause specific hazards for lung tumors, tumors (excluding lung tumors), and non-thymic lymphomas.

C

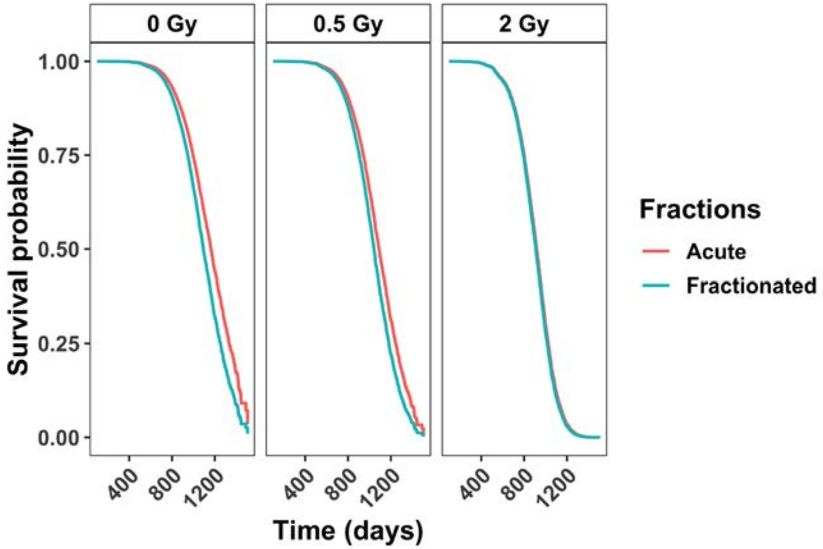

D

Term	estimate	Hazard Ratio (95% CI)	P-value
Sex(M)	-0.429	0.65 (0.60, 0.71)	**1.03E-25**
Fractionated	0.330	1.39 (1.25, 1.55)	**1.39E-09**
Total Dose	0.703	2.02 (0.93, 4.40)	0.077
Age first irradiated	0.003	1.003 (0.998, 1.009)	0.260
Fractionated:Total Dose	-0.136	0.87 (0.40, 1.91)	0.733

S5 Fig: CIF with a dose cutoff of 0.6 Gy

A

Variable	Parameter Estimate	Hazard Ratio (95% CI)	P-value
Sex(M)	-0.36	0.697 (0.615, 0.79)	**2.4E-08**
Total Dose	3.099	22.177 (10.52, 46.748)	**2.2E-16**
Age first irradiated	-0.002	0.998 (0.997, 0.999)	**1.1E-05**
Fractionated	-0.011	0.989 (0.847, 1.155)	0.89
Fractionated:Total Dose	-1.956	0.141 (0.058, 0.344)	**1.2E-05**

B

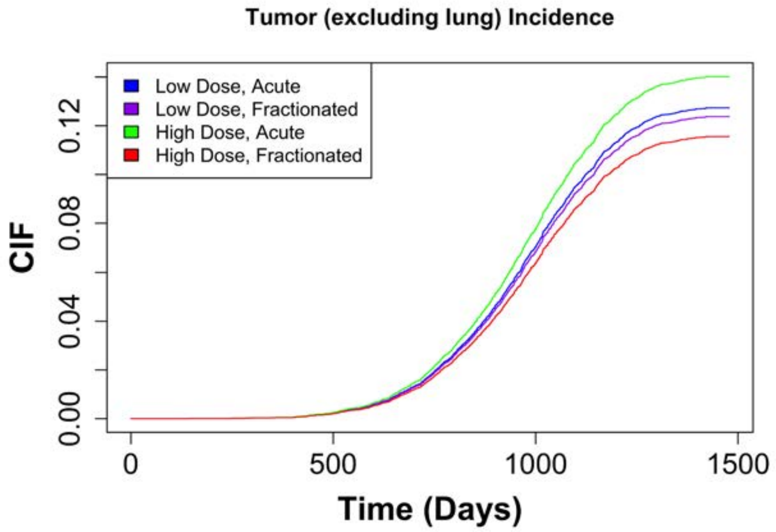

C

Variable	Parameter Estimate	Hazard Ratio (95% CI)	P-value
Sex(M)	1.187	3.276 (2.874, 3.734)	**0**
Total Dose	-0.392	0.676 (0.215, 2.127)	0.50
Age first irradiated	0.001	1.001 (1, 1.002)	**0.035**
Fractionated	-0.049	0.952 (0.793, 1.142)	0.600
Fractionated:Total Dose	0.942	2.566 (0.762, 8.637)	0.13

D

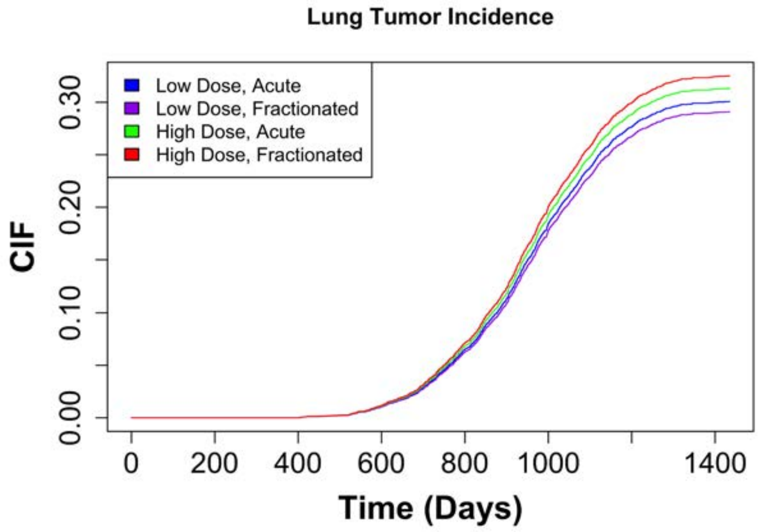

S5 Fig, continued: CIF with a dose cutoff of 0.6 Gy

E

Variable	Parameter Estimate	Hazard Ratio (95% CI)	P-value
Sex(M)	-0.362	0.696 (0.641, 0.757)	**0**
Total Dose	-1.223	0.294 (0.129, 0.67)	**3.5E-05**
Age first irradiated	0.001	1.001 (1, 1.002)	0.17
Fractionated	0.241	1.272 (1.144, 1.414)	**8.4E-06**
Fractionated:Total Dose	0.395	1.484 (0.605, 3.639)	0.39

F

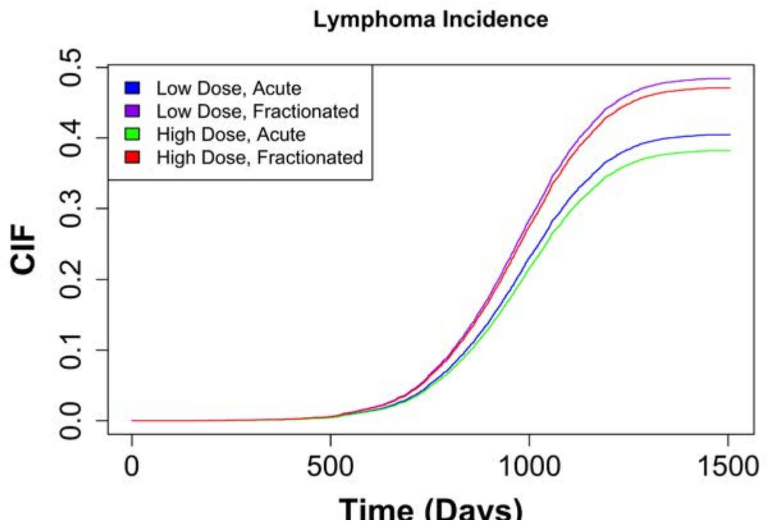

G

Variable	Parameter Estimate	Hazard Ratio (95% CI)	P-value
Sex(M)	-0.4	0.67 (0.573, 0.784)	**0**
Total Dose	-0.381	0.683 (0.251, 1.855)	0.45
Age first irradiated	0	1 (0.999, 1.001)	0.78
Fractionated	-0.527	0.59 (0.492, 0.708)	**0**
Fractionated:Total Dose	1.429	4.175 (1.305, 13.358)	**0.016**

H

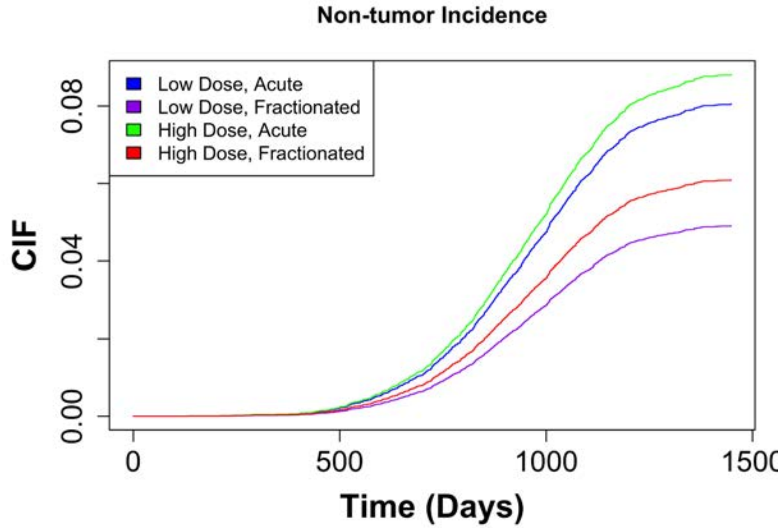

S4 Table: Main model output from Janus experiment 8 where mice were irradiated weekly until death

A

Variable	Parameter Estimate	Hazard Ratio (95% CI)	P-value
sexM	-0.114	0.89 (0.75, 1.06)	0.201
Dose Rate (0.148)	0.367	1.44 (1.15, 1.82)	**0.002**
Dose Rate (0.370)	0.972	2.64 (2.00, 3.49)	**7.13E-12**
Dose Rate (0.680)	1.901	6.69 (4.90, 9.14)	**7.07E-33**

B

Variable	Parameter Estimate	Hazard Ratio (95% CI)	P-value
sexM	-0.40	0.67 (0.56, 0.80)	**1.00E-05**
Dose Rate (0.0138)	0.72	2.06 (1.63, 2.60)	**1.20E-09**
Dose Rate (0.0349)	1.64	5.15 (3.83, 6.92)	**2.10E-27**
Dose Rate (0.0558)	1.87	6.47 (4.72, 8.88)	**3.94E-31**

S5 Table. Robustness testing output for the main model from Janus experiment 8 where mice were gamma irradiated weekly until death

A

Variable	Parameter Estimate	Hazard Ratio (95% CI)	P-value
Sex(M)	-0.115	0.89 (0.75, 1.06)	0.18
Dose Rate	2.814	16.67 (10.85, 25.6)	**8.10E-38**

B

Variable	Parameter Estimate	Hazard Ratio (95% CI)	P-value
Sex(M)	-0.114	0.89 (0.75, 1.06)	0.202
Dose Rate (0.148)	0.368	1.44 (1.15, 1.82)	**0.0018**
Dose Rate (0.370)	0.971	2.64 (2.00, 3.49)	**7.48E-12**
Dose Rate (0.680)	1.902	6.70 (4.91, 9.16)	**6.54E-33**
First Irrad	-0.003	1.00 (0.98, 1.01)	0.704

C

Variable	Parameter Estimate	Hazard Ratio (95% CI)	P-value
Sex(M)	-0.116	0.89 (0.75, 1.06)	0.18
Dose Rate	2.814	16.68 (10.86, 25.6)	**7.94E-38**
First Irrad	-0.003	0.997 (0.984, 1.011)	0.713

D

Variable	Parameter Estimate	Hazard Ratio (95% CI)	P-value
Dose Rate (0.148)	0.357	1.43 (1.13, 1.80)	**0.0025**
Dose Rate (0.148)	0.955	2.60 (1.96, 3.44)	**2.53E-11**
Dose Rate (0.370)	1.880	6.55 (4.78, 8.99)	**2.09E-31**

S6 Table: Robustness testing output for the main model from Janus experiment 8 where mice were neutron irradiated weekly until death

A

Variable	Parameter Estimate	Hazard Ratio (95% CI)	P-value
Sex(M)	-0.38	0.67 (0.57, 0.81)	**1.65E-05**
Dose Rate	32.5	1.33E14 (1.03E12, 1.73E16)	**3.44E-39**

B

Variable	Parameter Estimate	Hazard Ratio (95% CI)	P-value
Sex(M)	-0.40	0.67 (0.56, 0.80)	**1.05E-05**
Dose Rate (0.0138)	0.72	2.06 (1.63, 2.60)	**1.27E-09**
Dose Rate (0.0349)	1.64	5.15 (3.83, 6.93)	**2.00E-27**
Dose Rate (0.0558)	1.87	6.49 (4.73, 8.90)	**3.69E-31**
First Irrad	0.003	1.003 (0.99, 1.02)	0.665

C

Variable	Parameter Estimate	Hazard Ratio (95% CI)	P-value
sexM	-0.38	0.68 (0.57, 0.81)	**1.72E-05**
dose_rate	32.6	1.4E14 (1.1E12, 1.8E16)	**3.29E-39**
first_irrad	0.003	1.003 (0.99, 1.02)	0.62

D

Variable	Parameter Estimate	Hazard Ratio (95% CI)	P-value
Dose Rate (0.0138)	0.74	2.10 (1.66, 2.66)	**6.19E-10**
Dose Rate (0.0349)	1.62	5.05 (3.75, 6.79)	**1.16E-26**
Dose Rate (0.0558)	1.83	6.24 (4.55, 8.56)	**6.79E-30**

S6 Fig: KM curves for neutron and gamma irradiated JM8 mice to validate Cox PH model

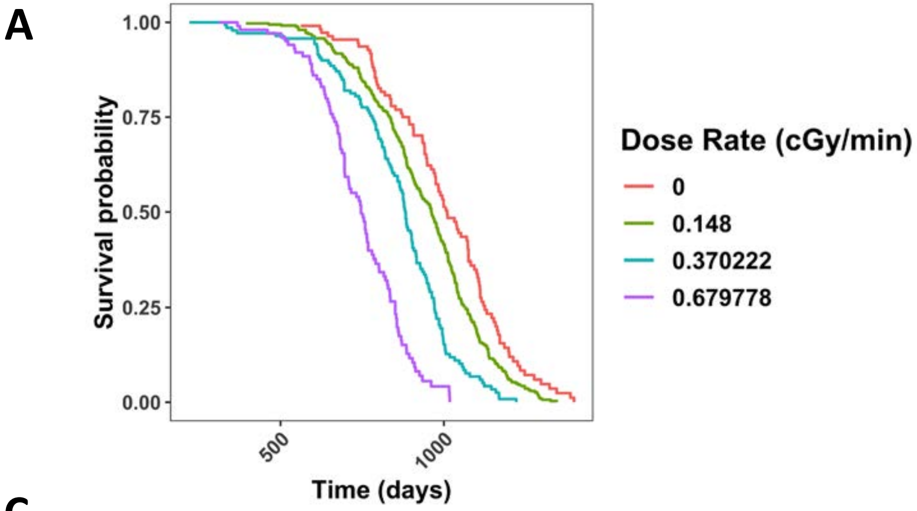

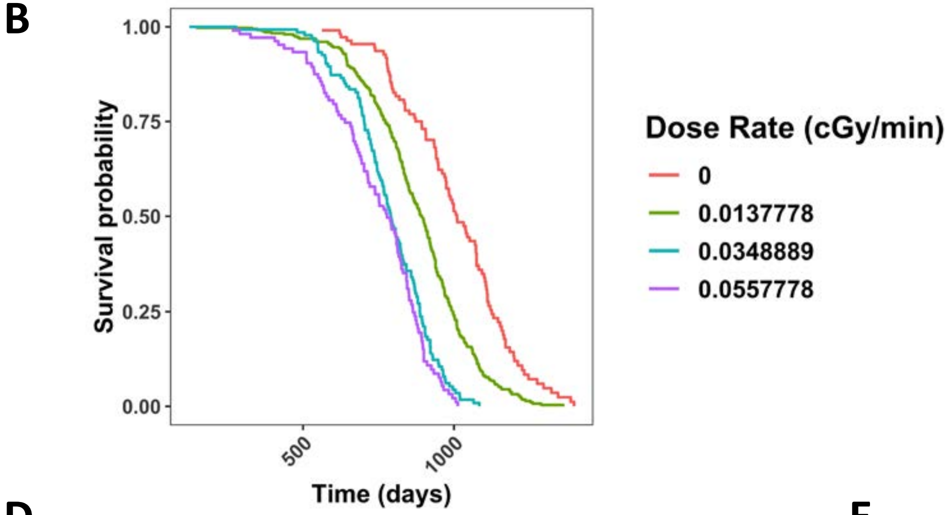

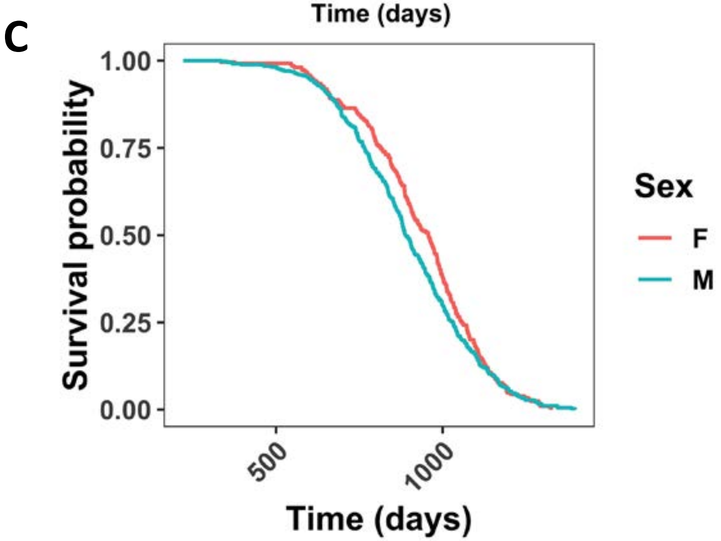

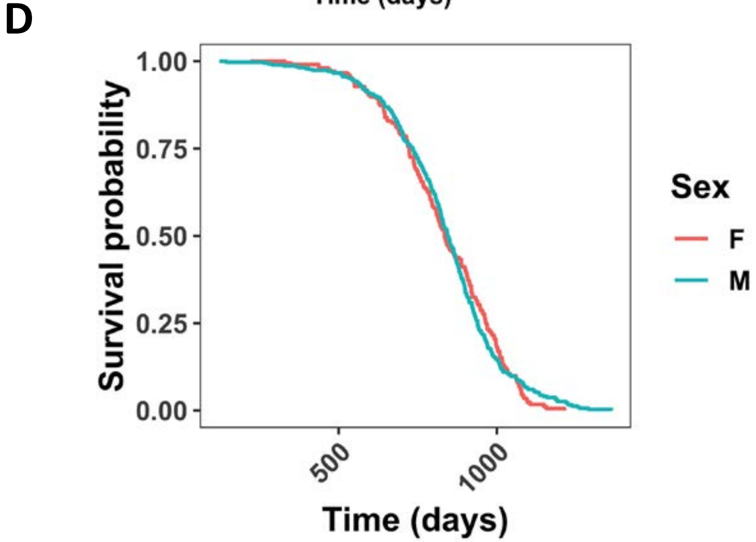

E

Term	P-value
SexM	0.38
Dose rate (0.138)	0.26
Dose rate (0.035)	0.99
Dose rate (0.056)	0.83
GLOBAL	0.62
